# Yellow Fever, Considered in Its Historical, Pathological, Etiological, and Therapeutical Relations

**Published:** 1855-11

**Authors:** 


ART. IV.—*Yellow Fever, considered in its Historical, Pathological, Etiological, and Therapeutical Relations. Including a Sketch of the Disease as it has occurred in Philadelphia from* 1699 *to* 1854. *With an Examination of the Connections between it and the Fevers known under the same name in other parts of Temperate, as well as in Tropical, Regions.* By R. LA ROCHE, M. D., Member of the American Philosophical Association; of the American Medical Association, etc., etc., etc. In two volumes. Blanchard & Lea, Philadelphia. 1855.

*The Cause and Prevention of Yellow Fever, contained in the Report of the Sanitary Commission of New Orleans.* By E. H. BARTON, A. M., M. D., Chairman of the Sanitary Commission, President of the Louisiana State Medical Society, and of the New Orleans Academy of Sciences, etc. Philadelphia: Lindsay & Blakiston. 1855.

When, a month since, we promised to devote more space than we could then spare to the second of these two importont works, we did not anticipate that we should so soon have the pleasure of examining the masterly work so long in preparation by Dr. La Roche. When we of the north, spared by a gracious Providence from the visitation of that terrible plague, the yellow fever, reflect upon the devastations it is even now inflicting upon the doomed cities of Virginia, it should arouse us from that apathy which we are too apt to feel concerning a disease which is not likely to come under our immediate notice. Even should we from selfish indolence forego the consideration of the morbid phenomena and the therapeutics of yellow fever, we should still recollect that in all its bearing upon sanitary police it comes home to us. The closest study of its causation serves only to prove that it, like our own epidemics of cholera and typhus, are but the rod of punishment for a wicked defiance or neglect of sanitary laws.

With the scope and argument of Dr. Barton's work our readers are already familiar. With that of Dr. La Roche it is desirable that they should be acquainted, if for no other reason than the example of its scholarship, research, and industry. In enumerating the bibliography of yellow fever, the author devotes forty-five pages of close print to the titles of books or pamphlets, which he has consulted. Following this we have nearly one hundred pages of preliminary observations, devoted to the medical topography of Philadelphia, and to the history of its various epidemics. In perusing this, with the map before us, we have solved one enigma, at least, which has hitherto puzzled us, viz., the presence of that rams horn, 'yclept Dock St., among the regular squares of the Quaker city. It is interesting, also, to recognize the fact that in the course of fourteen different epidemics, all have begun at nearly the same point, and infested the same localities.

As far back as the early epidemics we find two parties existing on the question of importation. As in New Orleans and elsewhere, a class of persons has always been found to ignore the putrescent sources of disease under their noses, to deny the deadly efficacy of filth and impure air, and to cast the whole murderous responsibility of a sweeping epidemic upon a single cotton bale or sugar hogshead. It is so easy to shoulder the blame upon Providence, so difficult to accuse ourselves of fatal negligence, that this doctrine will never want supporters. In the epidemic of 1793, Dr. Rush opened an issue with the College of Physicians (many of whom distinguished themselves by early flight from the epidemic) on the question of domestic origin. He told the truth fearlessly, and was warmly supported by the impetuous Charles Caldwell. Rush himself, at that time, advocated the doctrine of contagion coupled with that of domestic origin, a position which he afterward abandoned, and became a decided non-contagionist. And the number of pure contagionists has year by year diminished, until we now find but few leading minds in the profession willing to defend that doctrine.

It is gratifying to find that the two men most competent to speak upon the contagious character of yellow fever have reached the same conclusion by different routes. Dr. Barton, devoting himself to the study of atmospheric conditions, looks to them for his argument. Between the doctrine of causation by heat and humidity combined with local filth, and that of contagion, there is a natural and, to some extent, necessary antagonism. Dr. B. finds that one of these conditions being withdrawn, yellow fever ceases to exist; and with a pardonable air of triumph he drives home the argument in a few energetic sentences:

"No one pretends," he says, "that *sporadic* or *endemic* yellow fever is contagious? *Do these differ from epidemic yellow fever in their natures?* No one has the hardihood to make any such pretensions. A change of air which suddenly lowers the dew-point to near 58 degrees (here) if continued, puts an end to epidemic yellow fever; a crowded population may enter the city, occupy the houses, rooms, nay, the very beds, which lately reeked with yellow fever, yet not an instance, which can be attributed to contagion, occurs. The filth, the miasm, the terrene matters, are just as before. *But one change has occurred,* the connecting link, the combination, has been broken —*the meteorological element is wanting* and the *effects* are no longer present. Can anything be more conclusive? Where is the contagion now?"

Dr. La Roche makes it a matter of evidence. Collating from various sources the arguments for contagion, he presents them for the consideration of the student. Then, turning to the argument for non-contagion, he gives us 330 pages of proof arranged in such logical style, with every condition so fully discussed and fairly presented, that his is a stubborn mind who refuses his credence.

After giving an account of the *origin* of the doctrine of non-contagion, he supports it by adducing the number and ability of its believers; the age of the doctrine; the number of conversions from contagionism; the fact that contagionists are not particular in their choice of facts; that the disease appears at determinate periods of the year; that truly contagious diseases are not so regular in their period of outbreak; that yellow fever, in contradistinction to contagious diseases, is particularly under the influence of meteorological conditions, and is commonly associated with malarial fevers; that it absorbs other diseases, therein differing from contagious diseases; that it is a disease of hot weather and climate only; that is markedly influenced by atmospheric vicissitudes; that its malignity is increased by continued residence in infected localities; that its epidemic influence is felt by those who have avoided the sick; that it is often accompanied by unusual phenomena in vegetable life; that it is local in its habitation; that its area is circumscribed; that it is not communicated beyond that infected locality, and is not contagious in country districts; that its local origin is proved by sporadic cases; that it originates in low and impure localities, and so on for chapter after chapter, for hundreds of pages, he states his proposition, and goes on to prove it by an array of testimony such as we have never seen equalled in a medical work.

In considering *acclimatization*, Dr. La Roche does not differ from the established views. A prolonged residence in the yellow fever zone he considers as protective in the same manner that inhabitants of paludal districts are less liable to malarial disease than strangers. Second attacks are rare, and are evidently a process of acclimatization. The *temperaments* most liable to attack are those differing most widely from the bilious phlegmatic disposition, so peculiar to natives of tropical countries. The sanguine and robust form the victims. In *sex*, the female is less liable to attack from various causes which will suggest themselves. In childhood the same causes operate as a protection. *Race* has a marked influence. The pure negro is little liable to attack, the creole negro less so than the black of the north, and the liability to the disease, as well as its malignity, is increased with the amount of white blood in the individual. The dark complexioned Celt is also safer than the fair-haired, sanguine Saxon.

*Sleep* is mentioned as predisposing to attack—a fact allied to the known habits of malarial fevers everywhere. All *excesses* of diet, regimen, or exercise, as well as exposure to vicissitudes of weather, are also causes.

Passing for a moment to the consideration of light, electricity, and atmospheric pressure, we find that our author reckons the stimulus of a strong light as one of the causes, that little is known of the effect of variations in electrical conditions, and that so far as concerns atmospheric pressure the facts are contradictory. Dr. Barton, it is true, assigns a positive increase in the weight of the atmosphere as an epidemic cause. Our own limited observation confirms this view, but in many other cases the facts are precisely opposite.

We have purposely deferred to the close of our review, the discussion of that which we deem the most important element in the causation, not only of yellow fever, but of kindred malarial diseases—*the combination of heat and humidity.*

Dr. Barton, who must be considered the impersonation of the positive side of the question now arising, gives a *resumé* of the principles he deems established, as follows:

"1st. That the epidemic yellow fever has never occurred here* (at its commencement) but during a high dew-point (the minimum being upward of 74°.) In Savannah, last year, it was almost 2° less, and continued for some time.

"2d. That it has always ceased, as an epidemic, before it descended as low as 58°. In Savannah, last year, it terminated when it was a fraction less than 65°. In 1848, here, it ceased at about 1° higher, although the average of a series of years was, when it reached 62° 12′.

"3d. That at temperatures of the dew-point below these, sporadic or endemic yellow fever may occur, but it is not known to have existed here, with any certainty, as an epidemic, when the dew-point differed from that above stated.

"4th. That what is miscalled the CONTAGION OF YELLOW FEVER, or its liability to spread, exists *only with the first condition.*

"5th. That the main controlling influence, in all unhealthy situations, is MOISTURE, whether in cities, towns, countries, ships, or dwellings, although filth and heat are to be deemed correlative.

"6th. That MALARIA is not any one specific thing, but that all impurities of the air, and organic matter in decomposition, are liable to influence, inju-

---

* New Orleans.

riously, the organism, and particularly the worn out excreta of human beings may be so denominated, and are particularly incompatible with healthy action, and when in combination with the meteorological condition, may produce yellow fever."

Let us examine in what, how far, and with what reason, Dr. La Roche differs from these principles of causation.

The element of heat is conceded by all parties as essential to the generation of malaria, and the existence of yellow fever. As there is no difference of opinion as to its necessity between Drs. La Roche and Barton, it is unnecessary to pursue it further, except as Dr. La R. may give a higher importance to it than Dr. B. Neither does any difference manifest itself in their several views of malaria, Dr. La Roche using the word "infection" in a sense almost or quite identical with the "terrene causes" of Barton. His definition of infection is so restricted, and removes it so far from contagion, that we quote it, in the hope that it may become the fixed and settled meaning of the word.

"By infection, on the other hand, the reader will understand that power or poison which results from the decomposition of dead animal and vegetable substances, or other putrescent materials, if such exist, and through means of which a morbid state is induced in the system of individuals exposed to its action."

This "infection" is what Dr. La Roche considers the "efficient and immediate cause" of yellow fever. In discussing the influence of humidity, he admits the presence of a high hygrometric condition throughout the yellow fever zone, and the intimate relation existing between that condition and the production of disease. He states, however, that yellow fever does not *always* prevail when high heat and humidity are combined, and that it *does* prevail, sometimes, in dry seasons with an absence of these conditions. The first statement Dr. Barton can afford to admit, as it is possible that the terrene conditions were wanting. It should be recollected, moreover, that these terrene conditions can only exist in towns of considerable density of population. So far as we can speak with knowledge of the instances (Demarara, Pernambuco, and others) adduced by Dr. La Roche, as proving that high heat and humidity do not necessarily imply fever, they are small towns, and as little likely to afford the terrene cause as a valley in the country. Neither, so far as we can learn, does Dr. La Roche afford any positive information as to several very important points—such as the number of the foreign or unacclimatized population, or, except in two or three instances, the actual hygrometric condition of the atmosphere as derived by experiment. In all cases he assumes that insular position and tropical rains *must* produce high humidity. So far as insular position is concerned, it is a constant condition, and the inhabitants must be so accustomed to it as to deprive it of all force as a cause of disease; and in regard to the rains they are by no means a positive index. During the months of June, July and August, of 1855, in the city of Buffalo, there have fallen nearly fourteen inches of rain, while the atmosphere has been far drier, and the dew-point lower than during the drought of the summer of 1854, when only about four and a-half inches fell during the corresponding time. The immediate effect of a shower is to lessen the moisture of the air—it can only increase it indirectly by the succeeding accident of a hot sun, or a still air.

The second statement that it (yellow fever) does prevail, sometimes, with a positive absence of humidity, is, if correct, entirely fatal to Dr. Barton's theory, for we cannot in this instance fall back on the supposition that the terrene condition was at fault. We must, however, carefully except from this application the cases of Demerara, Charleston and New Orleans. To the former we have already objected as having a population by no means dense, and in all three of the towns cited the hygrometric condition existed; the only unfavorable circumstance being the fact that the dew-point was, though very high, very little or no higher in the epidemic, than in the healthy years. Now it has been repeatedly proven that, in the epidemic years, both New Orleans and Charleston had been subjected to great upheaval of the soil, and, moreover, it should be borne in mind that no one claims a high humidity *alone* to be a competent cause.

Thus far Dr. Barton's postulates remain untouched by the objections of Dr. La Roche. As it is admitted that only actual observation ought to determine the hygrometric condition, we can conscientiously assume that none of the instances as yet adduced ought to impair the value of Dr. B.'s theory.

It is in Philadelphia, however, that Dr. La Roche makes his strong point against the essential connection of high dew-point with fever. We shall follow his line of argument:

"The summer of 1699" (at Philadelphia, says Dr. La R.) was, as we have seen, one of the hottest, or, of course, one of the driest experienced—men died at harvest," &c. It does not follow, "of course," that it was "one of the driest," because it was one of the hottest. It might have been the counterpart of the year 1854, in Buffalo, and we have good reason for believing that men do not die at harvest in a dry heat. A high dew-point seems a necessity for *coup de soliel.*

"In 1762, it (yellow fever) prevailed after a hot and dry summer." He speaks, also, of the great epidemic year of 1793, as being very hot and dry. But in none of these, or in succeeding epidemics up to the year 1853, do we know anything positively about the real hygrometric condition of the atmosphere, except what Dr. Rush says of that of 1793, "that it was observed that while showers of rain lessened, moist or damp weather, without rain, increased it"—*i. e.* the fever.

It is, finally, upon Professor Kirkpatrick's tables of the climatic conditions of 1853, that Dr. La Roche relies to disprove the humid theory, or, at any rate, this is the only ground which in our opinion he ought to rest upon, inasmuch as it affords the only positive evidence given.

Prof. Kirkpatrick gives the mean dew-point of several months, in 1853, as follows: May, 49.3°; June, 54.2°; July, 57.2°; August, 59.3°, and September, 54.2°.

We admit at once that these are not epidemic dew-points, that they are not at all above what would be expected in ordinary seasons. But we propose to suggest an explanation for this also. Noticing that Prof. Kirkpatrick obtained lower dew-points than any other observer within our knowledge, we obtained (last spring) from him, through Dr. Hollingsworth, of the Phil. Med. Examiner, his method of reduction. It is simply that furnished with Mason's Hygrometer by the manufacturers. It directs the dew-point to be obtained by doubling the observed dryness, and adding one-third the difference, the sum to be subtracted from the temperature, the remainder being the dew-point. In common with all other observers with whom we have exchanged views, we believe this to be far too large a constant. But what is more to the purpose, we know that the constant used by Dr. Barton is the number 105, his method being comprised in multiplying the difference between the thermometers by 105, dividing the product by the temperature of evaporation, and deducting the quotient from the temperature of the air. We have at hand a very few of the details of Prof. Kirkpatrick's observations.* In one of them, for a period of ten days in June, 1853, (the year in question) Prof. K. gives the average temperature of the air at 2, P. M., as 89°, and that of evaporation as 77°. Let us compare his results with those Dr. Barton would obtain from the same data.

*Kirkpatrick's Formula.* The observed dryness (12°) multiplied by $2\frac{1}{3}$, gives the absolute dryness as 28°, which deducted from the temperature (89°) leaves the dew-point 61°.

---

* Taken from Prof. Blodget's paper on "Clinical Conditions of 1853," in N. Y. Journal of Medicine.

*Barton's Formula.* The observed dryness (12°) multiplied by the constant 105, gives a product of 1260, which divided by the temperature of evaporation (77°) gives an absolute dryness of 16.4°, which deducted from the temperature of the air gives a dew-point of 72.6°.

The only other detail we have of Prof. K.'s observations, is a period of six days in August, 1853. The average temperature of these days, at 2, P. M., was 91°, and the average wet bulb temperature was 80°: 91 — 80 = 11, the difference. The dew-point of this period by Prof. K.'s method would be 65.4°, while by Dr. Barton's it would be 76.6°.

It seems to us that our explanation solves the enigma. If it does not, we must concede that Philadelphia is an exception to all other climates.

Having advanced these reasons for believing that — contrary to Dr. La Roche's supposition — Philadelphia had a high dew-point in the only year really in question, we are in duty bound to give our reasons for preferring Dr. Barton's formula to that of Prof. Kirkpatrick's, for if Dr. B. should be wrong in this vital point his whole theory requires remodelling; while it is equally evident that no comparison should be instituted between the results of the two observers, unless they used the same formula.

Regnault's formula, derived from actual experiment, is the one adopted by all meteorologists, and especially sanctioned by the labors of such men as Blodget and Guyot. It is, however, so laborious, that it is not in common use, and the formula we have called "Barton's" (though we believe it originated with the American Philosophical Society, of which Dr. La Roche is a member) is largely substituted for it, as being the nearest convenient approximation to Regnault. The authority on which that used by Prof. K. is based, we have failed to learn. It approximates to Regnault's only at low temperatures — say about 40° — and becomes increasingly faulty as we reach higher temperatures.

We confess to a little pride in having picked a flaw in so faultless a work as La Roche on Yellow Fever. The evident fairness of the author's intention, the earnest desire for truth which marks every page, will give it a permanent influence in the medical world. And it is only on this question of the effect of combined heat and humidity that we differ from its author, and to this we have devoted the more attention, as it is a familiar subject to us, and one of vast importance in other epidemics than those of yellow fever.

To the medical scholar we commend both the works under notice as full of matter, which, though devoted to a special subject, is of wide application in almost every epidemic of hot weather. The coupling of the two together has enabled us to do that full justice to Dr. Barton's report which we were anxious to render, by placing it in a degree of antagonism with the labored work of the best medical scholar of our country. At the same time we have found abundant merit in that of Dr. La Roche. Upon the subjects of importation, contagion, and prevention, it points out the only safe course for the public or the profession.

We have purposely avoided any discussion of symptoms or treatment, which, though fully discussed by Dr. La R. would possess little interest for the majority of our readers, while at the same time we are saved the necessity of making an exhibit of our scanty information on those subjects.

Note.—Inasmuch as Prof. Kirkpatrick's formula is correct at 40°, and incorrect at higher temperatures, it is evident that any other fixed constant must also be faulty at given points. We make no apology, therefore, for appending the following letter from our friend Dr. Wm. Van Pelt, of Williamsville, Erie Co., N. Y., for whose kindness and mathematical skill we have been much and often indebted. It furnishes a sliding scale of constants, which must approximate very nearly to entire accuracy.—*Ed. Jour.*

*Dear Doctor,*—Excuse me for demanding so much of your attention. The following table of factors was established by actual observation made at the Greenwich Observatory, London, to convert wet bulb thermometer observations to Daniell's hygrometer dew-points. I think they are just what we want:

| Dry Bulb Thermometer. | Factors. | Dry Bulb Thermometer. | Factors. | Dry Bulb Thermometer. | Factors. | Dry Bulb Thermometer. | Factors. |
|---|---|---|---|---|---|---|---|
| 20° | 8.5 | 40° | 2.4 | 59° | 1.8 | 78° | 1.5 |
| 21 | 8.5 | 41 | 2.4 | 60 | 1.8 | 77 | 1.5 |
| 22 | 8.5 | 42 | 2.4 | 61 | 1.8 | 80 | 1.5 |
| 23 | 8.5 | 43 | 2.4 | 62 | 1.7 | 81 | 1.5 |
| 24 | 7.3 | 44 | 2.3 | 63 | 1.7 | 82 | 1.5 |
| 25 | 6.4 | 45 | 2.3 | 64 | 1.7 | 83 | 1.5 |
| 26 | 6.1 | 46 | 2.3 | 65 | 1.6 | 84 | 1.5 |
| 27 | 6.1 | 47 | 2.2 | 66 | 1.6 | 85 | 1.5 |
| 28 | 5.7 | 48 | 2.2 | 67 | 1.6 | 86 | 1.5 |
| 29 | 5.0 | 49 | 2.2 | 68 | 1.6 | 87 | 1.5 |
| 30 | 4.6 | 50 | 2.1 | 69 | 1.5 | 88 | 1.5 |
| 31 | 3.7 | 51 | 2.1 | 70 | 1.5 | 89 | 1.5 |
| 32 | 3.1 | 52 | 2.0 | 71 | 1.5 | 90 | 1.5 |
| 33 | 2.8 | 53 | 2.0 | 72 | 1.5 | 91 | 1.4 |
| 34 | 2.6 | 54 | 2.0 | 73 | 1.5 | 92 | 1.4 |
| 35 | 2.6 | 55 | 2 0 | 74 | 1.5 | 93 | 1.4 |
| 36 | 2.6 | 56 | 1.9 | 75 | 1.5 | 94 | 1.4 |
| 37 | 2.5 | 57 | 1.9 | 76 | 1.5 | 95 | 1.4 |
| 38 | 2.5 | 58 | 1.9 | 77 | 1.5 | 96 | 1.4 |
| 39 | 2.5 | | | | | | |

Ex.—The temp. of air being 80°, the wet bulb 68°, dew-point required.

80 — 68 = 12

12 × 1.6 = 19.2

80 — 19.2 = 60.8° Dew-point.

The above differs a little at certain temperatures, of course, when the barometer is higher or lower than usual, but for utility* it is near enough. I think, on the whole, the long formula I have used is the only one mathematically correct, but for use the factors vary enough to meet the requirements so far as meteorology is concerned.

Yours truly,

WM. VAN PELT.

